# Preparation of 3D printed silicon nitride bioceramics by microwave sintering

**DOI:** 10.1038/s41598-024-66942-w

**Published:** 2024-07-09

**Authors:** Xiaofeng Zeng, Coswald Stephen Sipaut, Noor Maizura Ismail, Yuandong Liu, Yan yan Farm, Bo Peng, Jiayu He

**Affiliations:** 1 Faculty of Engineering, University Malaysia Sabah, 88400 Kota Kinabalu, Sabah Malaysia; 2 Hengyang Kaixin Special Material Technology Co., Ltd., Hengyang, 421200 China; 3 School of Minerals Processing and Bioengineering, Central South University, Changsha, 410083 China

**Keywords:** Si_3_N_4_, Microwave sintering, Mechanical properties, Biocompatibility, 3D printing, Biotechnology, Engineering, Materials science

## Abstract

Silicon nitride (Si_3_N_4_) is a bioceramic material with potential applications. Customization and high reliability are the foundation for the widespread application of Si_3_N_4_ bioceramics. This study constructed a new microwave heating structure and successfully prepared 3D printed dense Si_3_N_4_ materials, overcoming the adverse effects of a large amount of 3D printed organic forming agents on degreasing and sintering processes, further improving the comprehensive performance of Si_3_N_4_ materials. Compared with control materials, the 3D printed Si_3_N_4_ materials by microwave sintering have the best mechanical performance: bending strength is 928 MPa, fracture toughness is 9.61 MPa·m^1/2^. Meanwhile, it has the best biocompatibility and antibacterial properties, and cells exhibit the best activity on the material surface. Research has shown that the excellent mechanical performance and biological activity of materials are mainly related to the high-quality degreasing, high cleanliness sintering environment, and high-quality liquid-phase sintering of materials in microwave environments.

## Introduction

Silicon nitride (Si_3_N_4_) is a non oxide ceramic that has been widely used in industry and has been studied for its use in the biomedical field since 1989^1–13^. The basic theory of using silicon nitride implant in the biomedical field is based on its excellent combination of mechanical performance, microstructure, and biological activity^4,6,8^. Porous silicon nitride implants have shown exciting results in the maxillofacial and spinal surgery^5,8^. However, Si_3_N_4_ material has high vickers hardness up to 15 GPa^2^, is not easy to process, has high customization costs, and a long preparation time cycle, so its market application is very limited. In addition, due to the high sintering temperature of Si_3_N_4_ materials, traditional sintering preparation methods such as gas pressure sintering (GPS) and hot press sintering (HPS) are influenced by various complex factors such as graphite atmosphere, heating environment, heat transfer mechanism, and high-temperature decomposition of Si_3_N_4_^14,15^. Its mechanical properties have not reached the ideal level, and its biocompatibility still fluctuates^6^. If the mechanical performance and biological activity of Si_3_N_4_ materials are further improved, the implants have great potential for application in areas that urgently need improvement, such as the heart and skull^11^. The performance of Si_3_N_4_ bioceramic materials still needs further improvement and verification.

Three-dimensional (3D) printing technology is an emerging digital model-based manufacturing technology that achieves object preparation through layer by layer construction. The 3D technology stands out for cost-effectiveness, adjustable speed for any design flexibility, positioning itself. It is a cutting-edge hot spot in the study of advanced material forming and preparation technology. It has great potential for application in the field of inorganic non-metallic materials^16–22^. In recent research, 3D printing can solve the problem of customizing silicon nitride bioceramic materials^12,21,23^. However, the organic content of 3D printing slurry is high, with DLP resin content exceeding 50 vol%^21,23^ and FDM organic content also reaching 47 vol%^9,12^, which is prone to defects such as degreasing, cracking, and low sintering density. This is currently a major bottleneck problem hindering the preparation of dense silicon nitride bioceramics by 3D printing.

Microwave sintering (MS) is a method of achieving densification by coupling the special wavelength band of microwave with the basic microstructure of materials to generate heat, and the dielectric loss of materials heats them up to the sintering temperature as a whole. It is an efficient and energy-saving sintering method, which has many advantages such as selective self-heating, uniform and rapid heating, reduced sintering temperature, and no pollution^24–29^. If it is applied to the preparation of Si_3_N_4_ materials, it is of great significance to improve the degreasing effect, production efficiency, sintering environment of Si_3_N_4_ materials, reduce the sintering temperature of Si_3_N_4_, inhibit the decomposition of Si_3_N_4_, and improve the mechanical performance and biological activity of the Si_3_N_4_ materials. However, due to the poor absorption performance of Si_3_N_4_ materials, they cannot be effectively heated quickly, and the high sintering temperature of Si_3_N_4_ materials makes it impossible for sintered insulation materials to directly withstand its temperature^30,31^. Therefore, a new MS structures suitable for degreasing and sintering of Si_3_N_4_ urgently need to be established. In recent years, there are few research reports on microwave sintering of dense Si_3_N_4_ materials.

This research prepared dense Si_3_N_4_ bioceramics using fused deposition molding (FDM) printing method, microwave rapid degreasing and sintering method. Their mechanical performance and biological activity were tested, and compared with GPS Si_3_N_4_, and HPS Si_3_N_4_, Alumina, Titanium alloy, and PEEK. The influence of microwave heating method on the rapid and safe degreasing, crystal structure growth, mechanical performance, and biocompatibility of 3D printed silicon nitride materials were discussed, and their mechanisms were simulated and explained. Finally, positive and feasible results were obtained for the application of microwave sintered 3D printed dense silicon nitride bioceramic materials in the biomedical field.

## Materials and methods

### Preparation of Si_3_N_4_ bioceramics

**Figure 1:**
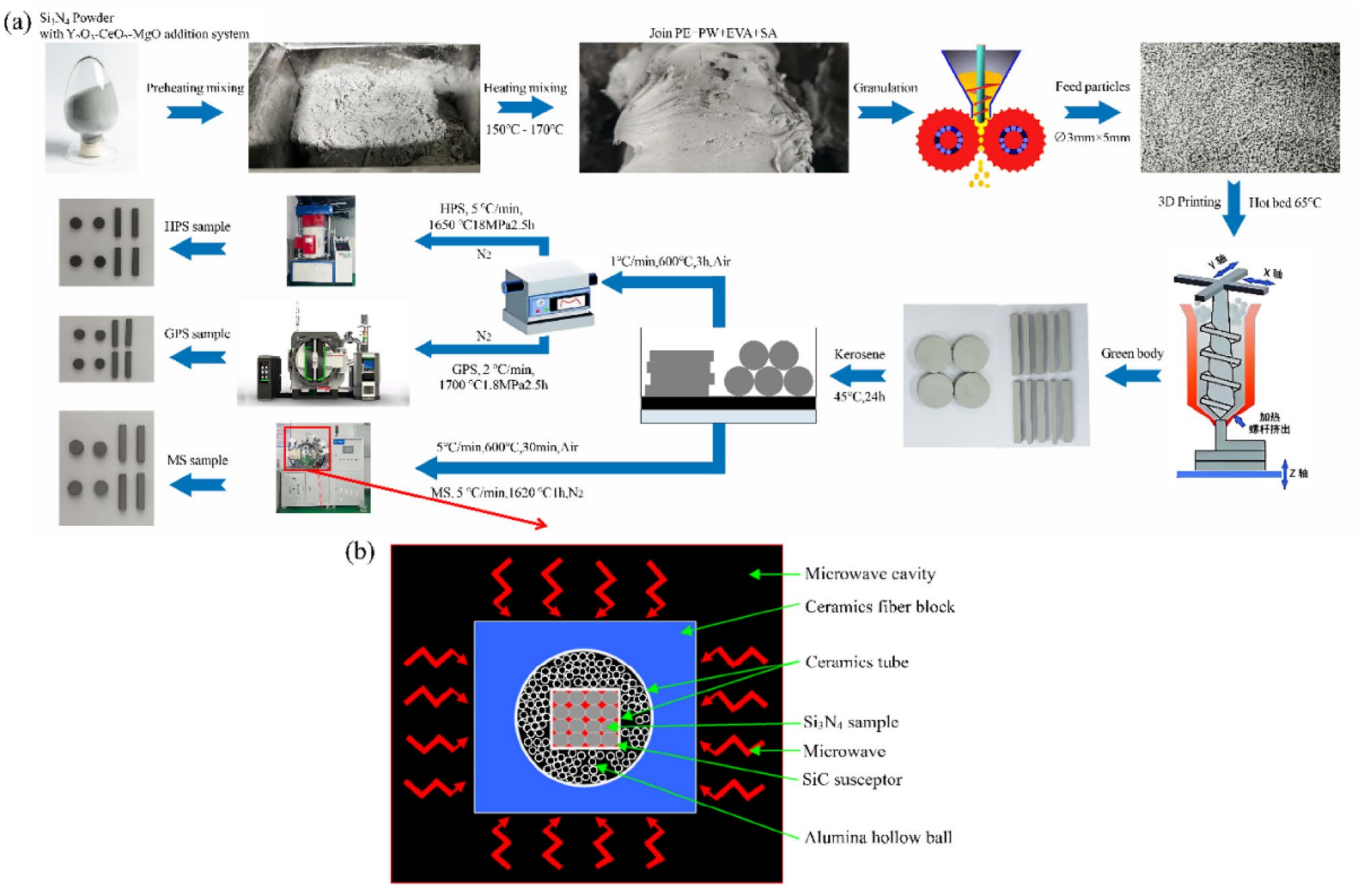
(**a**) Preparation process of 3D printed Si_3_N_4_ bioceramics samples; (**b**) The new MS structure diagram constructed in this research.

The preparation process of 3D printed Si_3_N_4_ bioceramics was shown in Fig. 1a. Similar to previous research^12^, the raw material used in this study was Si_3_N_4_ powder (Ube, SN-E10, Japan, D_50_ = 0.65 μm, SBET = 8.7 m^2^/g), with Y_2_O_3_, CeO_2_, and MgO as sintering aids. Mix ceramic powder and organic adhesives (polyethylene, paraffin, ethylene vinyl acetate copolymer, stearic acid) in a double roller mixer at a mixing temperature of 150–170 °C for 55 min to form a slurry. The overall volume fraction of ceramic powder in the slurry is 53%. 3D test samples were prepared and printed using a screw extrusion FDM printer (UP-R200, Shenzhen Shenghua 3D Technology Co., Ltd.). The 3D test samples were prepared in strip shape of 50 mm × 6 mm × 4 mm and circular shape with dimension of Φ13 mm × 4 mm using a “single line” filling path. The printed samples degreased in a 45 °C kerosene solvent for 24 h, and then dried it in a 45 °C hot air oven for 16 h.

The next program is degreasing, with the aim of safely removing large amounts of organic matter from 3D printed samples. For the preparation of GPS and HPS samples, in order to reliably degrease the samples, the dried bodies were placed in a hot degreasing furnace and heated in the air at a heating rate of 1 °C/min to 600 °C. During the degreasing process, the samples were kept at 200 °C for 1 h, followed by 300 °C for 2 h, and lastly 600 °C for 3 h, respectively. For the GPS method, the hot degreased bodies were placed in a gas pressure sintering furnace (5518, 6 MPa, Xiangtan Xinyun Technology Co., Ltd.) and heated to 1700 °C at a heating rate of 2 °C/min under nitrogen pressure of 1.8 MPa, with insulation for 2.5 h. For the HPS method, the hot degreased bodies were placed in a hot press sintering furnace (1800 °C, 25 MPa, Hunan Jinteke Technology Co., Ltd.) and heated to 1650 °C at a heating rate of 5 °C/min under nitrogen atmosphere (atmospheric pressure) and a mechanical pressure of 18 MPa, with insulation for 2.5 h.

For the MS method, the dried bodies were placed in a gas microwave sintering furnace (2.45 GHz, 6 kW, Hunan Huaye Microwave Technology Co., Ltd.). Firstly, microwave heating degreasing was carried out in the air at a heating rate of 5 °C/min to 600 °C, followed by insulation at 200 °C for 15 min, 300 °C for 30 min, and 600 °C for 30 min. Then, gas was replaced (microwave heating was suspended), and heating was continued at a heating rate of 5 °C/min under a nitrogen atmosphere (atmospheric pressure) to 1620 °C, with insulation for 1 h. The new MS structure is shown in Fig. 1b. The system consists of four 1.5 kW 2.45 GHz microwave sources and two ceramic (Alumina) tubes. The outer ceramic tube was inserted into the cavity and surrounded by alumina ceramic fiber blocks. The internal ceramic tube was inserted into the outer one. Fill high-purity alumina hollow balls between the inner and outer alumina ceramic tubes. The materials were placed inside the internal ceramic tube and putted on the SiC susceptor. Due to the combination of double alumina tube and high-purity alumina hollow ball structure, the system was capable of achieving temperatures above 1600 °C within a short time, and maintain the high temperature for a longer period of time.

After sintering, the bioceramics samples were ground using diamond grinding tool (Diamond particles of 180 mesh) to produce strip shaped (3 mm × 4 mm × 36 mm) and circular (Φ10 × 3 mm) specimens with a surface finish of 0.6 μm. The control material samples with the same specifications include Alumina (99%, Foshan Haocai New Material Technology Co., Ltd.), Titanium alloy (CT4, Hebei Bo Titanium Metal Materials Co., Ltd.), and PEEK (Faint yellow, Shenzhen Caocheng Plastic Materials Co., Ltd.).

### Physical characterization of samples

The density of both treated samples was measured by the Archimedes drainage method. Vickers hardness of samples was measured by using a hardness tester, applying a test force of 98.1 N (10 kg) to materials using an indenter, and holding it for 15 s to obtain hardness data. The three-point bending strength and compressive strength of the long strip specimen were tested with a universal material testing machine (Instron Model 5928, USA). The elastic modulus is calculated by formula (1) (ICS 81. 060. 20: Q 32):

1 $$E = \frac{{3L(P_{2} - P_{1} )}}{{2bh^{2} \left( {\varepsilon_{2} - \varepsilon_{1} } \right)}} \times 10^{ - 3}$$

*P*_*1*_ and *P*_*2*_ are the initial and final loads of the material loaded within a linear range, respectively. *L* is the distance between the sample supports, *b* is the width of the sample, *h* is the thickness of the sample, and *ε*_*1*_ and *ε*_*2*_ are the strain at the mid span of the sample corresponding to *P*_*1*_ and *P*_*2*_, respectively. The variables on the formula (1) can be measured by the universal material testing machine (Instron Model 5928, USA). The fracture toughness of samples was tested using the method of unilateral pre-cracking. SEM (TESCAN Bmo, s.r.o., TESCAN MIRA, Brno, Czech Republic) was used to observe the microstructure of Si_3_N_4_ bioceramics samples. Samples phase analysis was performed by XRD (40 kV, 40 mA, D/MAX-RBX, Rigaku, Osaka, Japan), with a scanning speed of 5°/min and a scanning range of 5–80°.

### Cytotoxicity test

Take MC3T3-E1 cells (Wuhan Pricella Biotechnology Co., Ltd., Hubei, China) cultured to the third generation, digest with 0.25% trypsin, and resuspend evenly α In MEM culture medium, adjust the cell concentration to 3 × 10^4^/mL using a blood cell counting plate. Place the material sample in a culture dish, inoculate the above cell suspension for in vitro cell culture, and culture it in a 37 °C incubator. At three time points on the 1st, 3rd, and 5th days, discard the original culture medium, add CCK-8 reagent and culture medium, place them in the dark environment to incubate for 2 h, and take them out. Use a fully automatic multifunctional microplate reader (Biotek Instruments.Inc Epoch™) to measure the absorbance value (OD value) of each well at 450 nm for the culture medium containing CCK-8 in the culture dish, and collect the detection data.

### Evaluation of antibacterial property

Antibacterial properties of samples against *Staphylococcus aureus* (*S. aureus*, Beijing Biobw Biotechnology Co., Ltd., Beijing, China) and E. coli (E. coli, Beijing Biobw Biotechnology Co., Ltd., Beijing, China) were determined by colony count method. Luria Bertani broth was used for culturing *S. aureus* (ATCC 25,923) and E. coli (ATCC 25,922). The microbiological culture was inoculated to the sample specimen and incubate at 37 °C for 24 h. A certain amount of PBS was used to strongly clean the sample and separate microorganisms from the material. Sterilized PBS was used to dilute the eluent. A total of 100 μL of diluted eluent was added to LB broth agar medium. This was then incubated at 37 °C for 24 h, and the number of colonies counted.

### Cell adhesion and morphology

MC3T3-E1 cells were seeded on the discs at a density of 4 × 10^4^ cells per well in 24-well plates and cultured for 1 day. The samples were then stained with 2-(4-Amidinophenyl)-6-indole-

-carbamidine dihydrochloride (DAPI, Beyotime Biotech, Shanghai, China) and Actin-Tracker Red-Rhodamine (Beyotime Biotech, Shanghai, China) to observe the cell morphology using confocal laser scanning microscope (CLSM, Zessi, LSM800, Oberkochen, Germany). The morphology of the adhered cells on the discs was also observed by SEM (TESCAN Bmo, s.r.o., TESCAN MIRA, Brno, Czech Republic) after dehydrating the samples in gradient ethanol solutions (30, 50, 70, 85, 90, and 100%, respectively) for 10 min and drying at the critical point.

### Statistical analysis

Each experiment was repeated three times for statistical analysis. The mean ± standard deviation is used to represent the experimental results. Use statistical product and service solution 26.0 software (SPSS 26.0, IBM, New York, USA) to conduct student one-way analysis of variance (ANOVA) on the data *P < 0.05 indicates statistical significance.

## Results

### Characterization of materials

**Figure 2:**
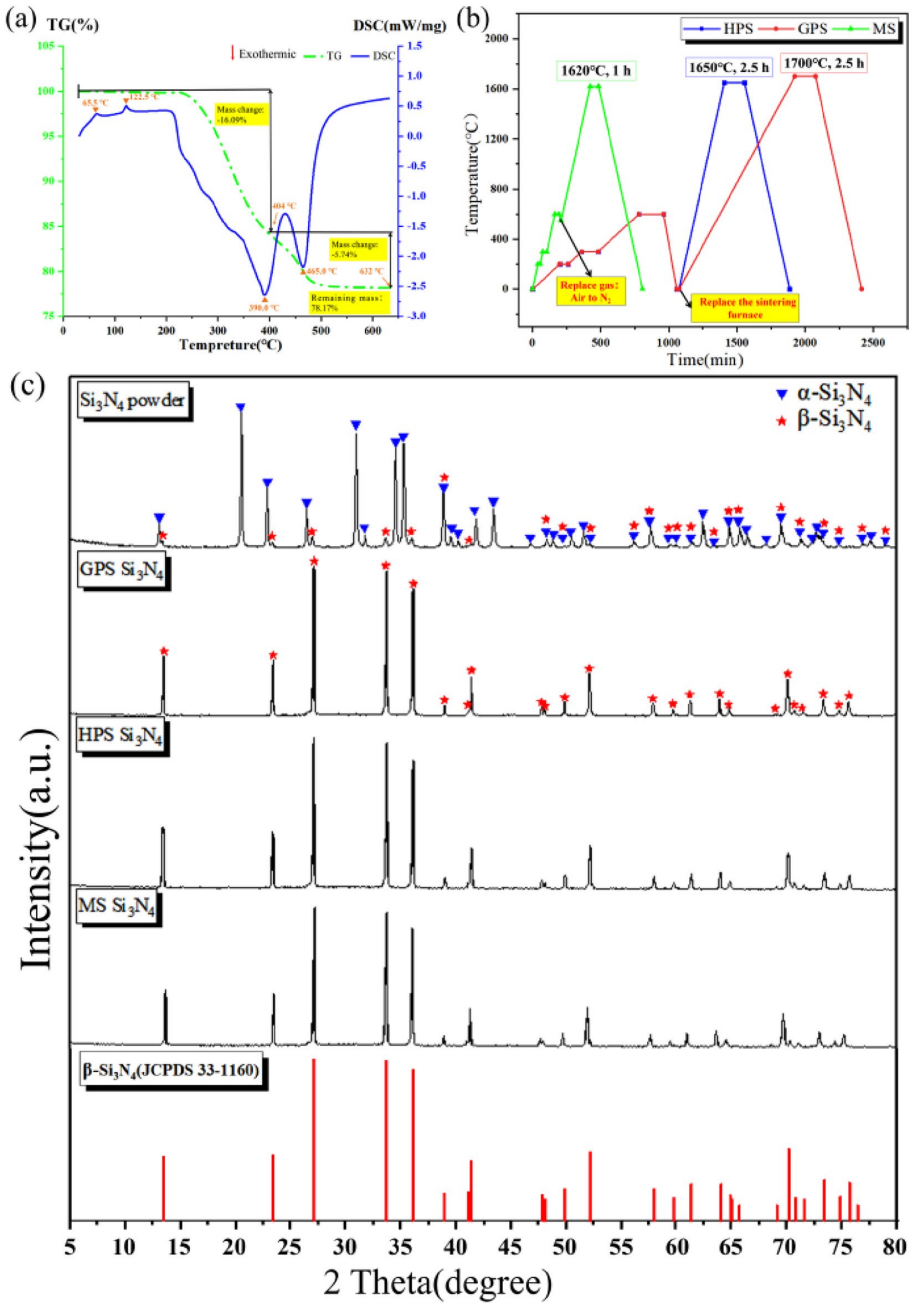
(**a**) The TG-DSC curve of 3D printed Si_3_N_4_ body; (**b**) The heating curves of 3D printed MS, GPS, and HPS Si_3_N_4_ samples; (**c**) Comparison of XRD between 3D printed MS, GPS and HPS samples, all peaks correspond to those of a reference β-Si_3_N_4_ (JCPDS 33-1160).

The 3D printed Si_3_N_4_ billet prepared in this study contains a large amount of organic matter. The TG-DSC curve in the air atmosphere is shown in the Fig. 2a. It can be seen that the significant weightlessness starts from above 200 °C and ends completely at around 600 °C. The total weight of the billet has been reduced to 78.17%, which is consistent with the solid content of the slurry. So, the mixing temperature of slurry in this research is suitable from 150 to 170 °C, and the mixing operation within this temperature range will not affect the organic matter content in the printed material. And the organic matter can be completely removed by holding at 600 °C for 3 h. In addition, a significant exothermic peak appeared after 390 °C, which was caused by the further exothermic reaction between the residual carbon generated after organic matter cracking and oxygen in the air. These residual carbons contribute to the absorption of microwaves for auxiliary heating at 400– 500 °C in this study, achieving uniform heating.

Figure 2b shows the heating curves of 3D printed MS, GPS, and HPS Si_3_N_4_ samples. Although there is a large amount of organic matter in the 3D printed Si_3_N_4_ body, due to the characteristics of microwave heating, the material itself absorbs microwave energy for heating. The MS curve has a fast-heating speed, short insulation time, high efficiency, and does not require equipment replacement, allowing for continuous degreasing and sintering. However, GPS and HPS equipment cannot be heated to 600 °C in the air to remove residual carbon due to their graphite heating element and carbon insulation materials. Therefore, degreasing and sintering need to be carried out in different equipment. In addition, due to the fact that the degreasing furnace also belongs to radiation heating, the heating uniformity is poor, and the removal of a large amount of organic matter requires slow degreasing. It is also important to note that the low strength green body after degreasing needs to be carefully transported, otherwise it may cause damage during transportation, resulting in a significant decrease in preparation efficiency.

Figure 2c shows the XRD of 3D printed MS, GPS and HPS Si_3_N_4_ samples. Figure 2c evidences that the α-Si_3_N_4_ crystal phase has completed transformation and transformed into β-Si_3_N_4_ after densification sintering for samples of MS Si_3_N_4_, GPS Si_3_N_4_, or HPS Si_3_N_4_. The main characteristic peaks of β-Si_3_N_4_ at 2θ = 27.2°, 33.8°, and 36.2° appear in the XRD spectra, and all peaks correspond to those of the reference β-Si_3_N_4_ (JCPDS 33-1160). Due to the Y_2_O_3_-CeO_2_-MgO sintering aid system, the temperature point at which liquid phase appears in silicon nitride materials is approximately 1500 °C^32,33^. In this research, the higher sintering temperatures resulted in more pronounced liquid phase effects and more complete crystal growth^12^. Moreover, at the high temperatures, sintering aids gradually evaporate or exist in the silicon nitride lattice, resulting in no relevant crystal phase display in XRD^34,35^.

**Figure 3:**
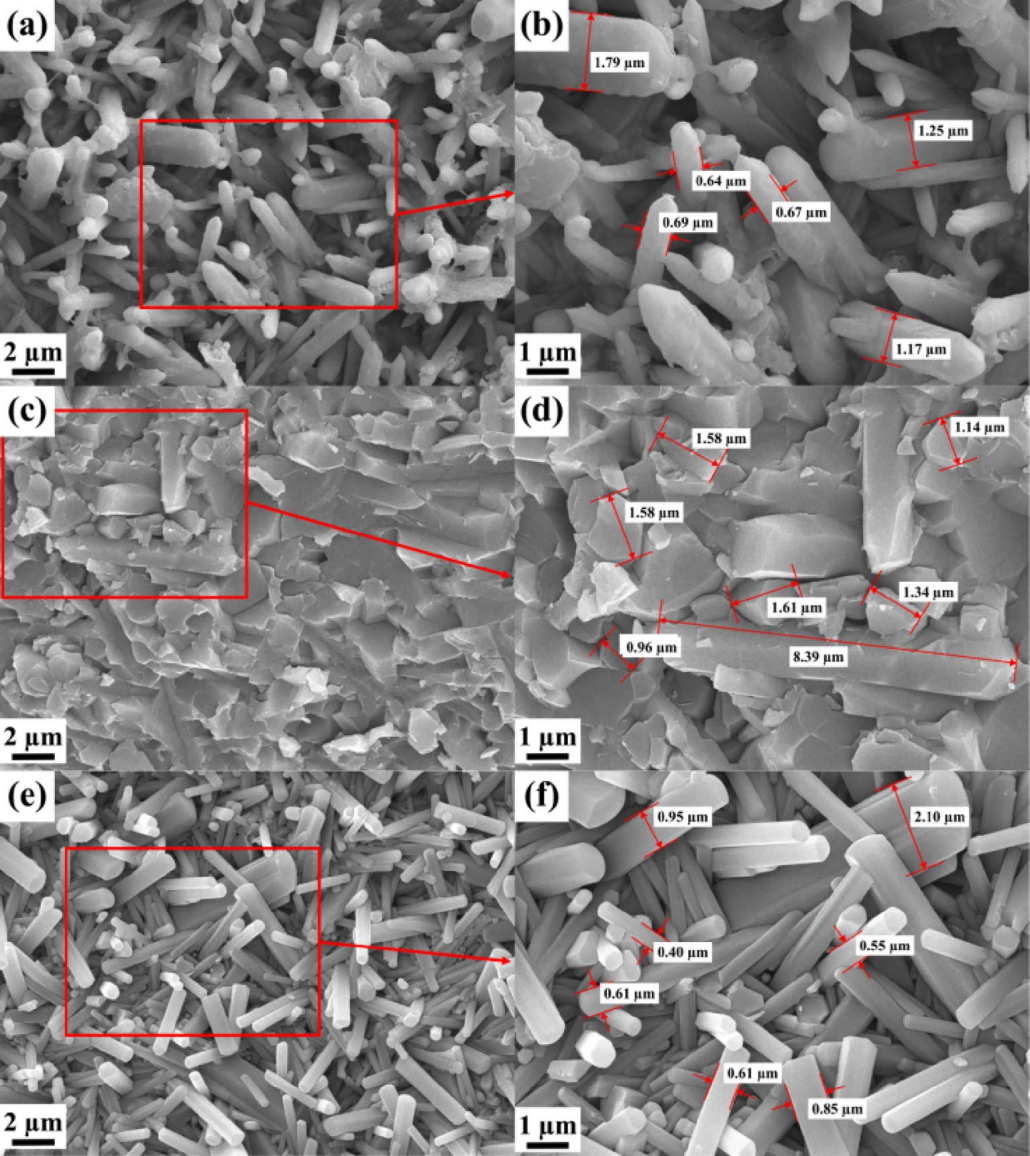
Microscopic morphology of the 3D printed MS, GPS and HPS samples. (**a**) The surface SEM of GPS Si_3_N_4_ (5.0 kx); (**b**) The surface SEM of GPS Si_3_N_4_ (10.0 kx); (**c**) The surface SEM of HPS Si_3_N_4_ (5.0 kx); (**d**) The surface SEM of HPS Si_3_N_4_ (10.0 kx); (**e**) The surface SEM of MS Si_3_N_4_ (5.0 kx); (**f**) The surface SEM of MS Si_3_N_4_ (10.0 kx).

To further study the mechanism of material action, SEM microscopic morphology analysis was conducted on the surface of the 3D printed Si_3_N_4_ sample of MS, GPS, HPS, as shown in Fig. 3. As shown in Fig. 3a, b, the crystal boundaries of the GPS Si_3_N_4_ sample are not very clear, with a small number of impurities between the grain boundaries. The crystal growth is incomplete, and the crystal shape is basically long columnar, but there are many connected crystals, indicating that there is still residual liquid phase. The crystal diameter is relatively large, with a typical crystal diameter of 0.6–0.8 μm and a small aspect ratio of about 3– 5. As shown in Fig. 3c, d, the crystal boundaries of the HPS Si_3_N_4_ sample are not clear. There are many impurities between the grain boundaries. The crystal growth is incomplete, and the crystal shape is irregular, making it difficult to distinguish obvious long columnar crystals. The connection between the crystals is severe, indicating that there is still a large residual liquid phase. The crystal size is relatively large, with a typical crystal diameter of 1.0–2.0 μm. Because the crystals are basically in an intersecting state, the aspect ratio cannot be estimated. From Fig. 3e, f, it can be seen that the crystal boundaries of MS Si_3_N_4_ sample are clear. There are basically no residual liquid impurities between the grain boundaries. The crystal growth is complete, with clear diamond angles and a regular crystal shape of long columnar crystals. The crystal diameter is small, with a typical crystal diameter of 0.4–0.6 μm and a large aspect ratio of about 5–10. Compared with MS sintering technology, in the case of GPS and HPS sintering technology, there are obvious impurities in the silicon nitride grain boundaries. These impurities are high-temperature liquid phase substances generated by the joint action of silicon nitride materials and additives during the sintering process^9,14^. These high-temperature liquid phase substances play a crucial role in the transformation of silicon nitride crystal phase at high temperatures. After the completion of the transformation, they are ultimately retained in the silicon nitride grain boundaries due to the influence of sintering methods or the degree of liquid phase action. These grain boundary phase substances are brittle and have a significant negative impact on the mechanical properties of silicon nitride sintered materials^9,14,15^. From the microstructure shown in Fig. 3, different fracture modes can also be observed, indicating significant differences in crystal integrity and strength of silicon nitride materials under different sintering methods. After the GPS sample fractures, it basically presents a relatively complete crystal morphology. Although there is a small amount of liquid phase adhesion, the crystal strength is high and not easily damaged; After the fracture of the HPS sample, it basically presents a crystal damage morphology, with a lot of liquid phase substances at the grain boundaries, resulting in abnormal crystal growth and high brittleness^14,15^; After the MS sample fractures, it presents a complete crystal morphology with almost no liquid phase material in the grain boundaries. The crystal has high strength and is not easily damaged. This material relies on the intersection of intact crystals to achieve high mechanical strength^12^.

### Mechanical properties of materials

Table 1 shows the comparison of mechanical performance between 3D printed Si_3_N_4_ samples with different sintering method and Al_2_O_3_, Ti-alloy, PEEK, and Cortical bone. As shown in Table 1, the density of MS Si_3_N_4_ sample is slightly lower than that of GPS Si_3_N_4_ samples and HPS Si_3_N_4_ samples, but the difference is not significant, with an average density of 3.23 g·cm^−3^, which is at an intermediate level compared to other comparative materials. In terms of elastic modulus, MS Si_3_N_4_ sample is not significantly different from GPS Si_3_N_4_ and HPS Si_3_N_4_, and belongs to the intermediate level compared to other comparative materials. The MS Si_3_N_4_ samples exhibits exllent mechanical performance: bending strength is 928 MPa, fracture toughness is 9.61 MPa·m^1/2^, vickers hardness is 16.2 GPa, compressive strength is 3215 MPa. There are also significant advantages compared to the other materials: Ti-alloy, Al_2_O_3_, PEEK, and Cortical bone.

**Table 1:** Comparison of mechanical properties between 3D printed Si_3_N_4_ samples with different sintering method and Al_2_O_3_, Ti-alloy, PEEK, and Cortical bone; properties of cortical bone are shown for reference^5^.

Property	GPS Si_3_N_4_	HPS Si_3_N_4_	MS Si_3_N_4_	Ti-alloy	Al_2_O_3_	PEEK	Cortical bone
Density (g·cm^−3^)	3.25 ± 0.02	3.26 ± 0.02	**3.23 ± 0.02**	4.43 ± 0.02	3.97 ± 0.03	1.29 ± 0.01	1.85
Elastic modulus (GPa)	314 ± 10	320 ± 8	**318** ± **7**	110 ± 7	435 ± 20	4 ± 1	8–12
Flexural (MPa)	803 ± 30	825 ± 35	**928 ± 30**	–	420 ± 50	170 ± 10	–
Fracture toughness (MPa·m^1/2^)	8.86 ± 0.41	8.53 ± 0.42	**9.61 ± 0.31**	75 ± 2	4.51 ± 0.5	–	–
Vickers hardness (GPa)	15.1 ± 0.3	15.3 ± 0.3	**16.2** ± **0.2**	3.4 ± 0.1	19.1 ± 1	–	–
Compressive (MPa)	2725 ± 201	2817 ± 183	**3215 ± 170**	958 ± 79	2350 ± 255	135 ± 15	130–190

### Cytotoxicity determination

**Figure 4:**
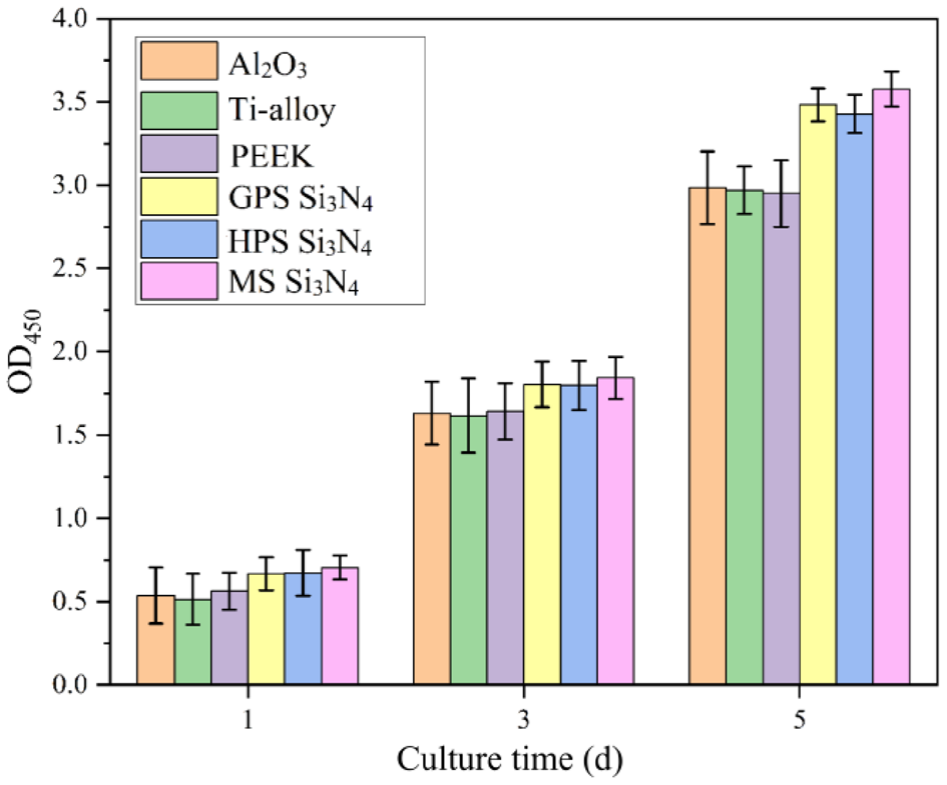
Comparison of cytotoxicity between 3D printed Si_3_N_4_ samples with different sintering method and Al_2_O_3_, Ti-alloy, PEEK.

The cytotoxicity test results of MS Si_3_N_4_, GPS Si_3_N_4_ and HPS Si_3_N_4_ are shown in Fig. 4. During the cell culture time, cells can grow normally on different sintering processes of Si_3_N_4_ bioceramics and control samples, and the cell viability on Si_3_N_4_ sample is obviously higher than that on the control materials such as Al_2_O_3_, Ti-alloy, and PEEK. The difference in cytotoxicity of Si_3_N_4_ samples prepared by different sintering processes is not significant. From the results data, the OD_450_ value of MS Si_3_N_4_ is 3.51, the OD_450_ value of HPS Si_3_N_4_ is 3.40, and the OD_450_ value of GPS Si_3_N_4_ is 3.44. The larger the OD_450_ value, the lower its cytotoxicity. Therefore, in terms of cytotoxicity, MS Si_3_N_4_ < GPS Si_3_N_4_ < HPS Si_3_N_4_. The results indicate that the Si_3_N_4_ sample prepared by MS process has the lowest cytotoxicity.

### Evaluation of antibacterial property

**Figure 5:**
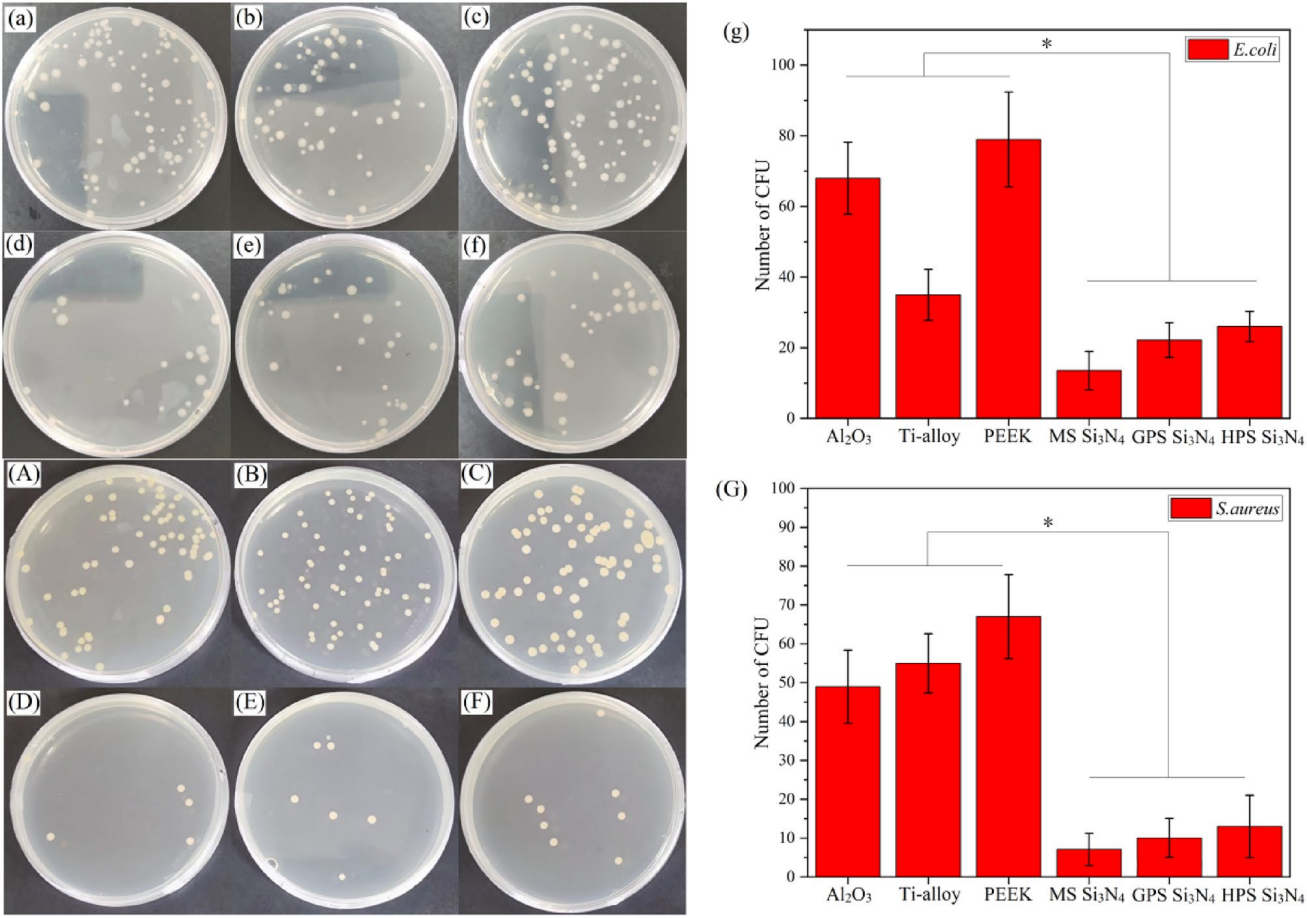
Comparison of antibacterial properties between 3D printed Si_3_N_4_ samples with different sintering method on *S. Aureus* and E. coli. Displayed the E. coli colony plates of Si_3_N_4_ samples prepared by different sintering processes: (**a**) Al_2_O_3_; (**b**) Ti-alloy; (**c**) PEEK; (**d**) MS Si_3_N_4_; (**e**) GPS Si_3_N_4_; (**f**) HPS Si_3_N_4_; (**g**) Bar chart of antibacterial activity of Si_3_N_4_ samples prepared by different sintering processes and control samples on E. coli (*P < 0.05). Displayed the colony plates of *S. aureus* prepared by different sintering processes for Si_3_N_4_ samples: (**A**) Al_2_O_3_; (**B**) Ti-alloy; (**C**) PEEK; (**D**) MS Si_3_N_4_; (**E**) GPS Si_3_N_4_; (**F**) HPS Si_3_N_4_; (**G**) Bar chart of antibacterial activity of Si_3_N_4_ samples prepared by different sintering processes and control samples on *S. aureus* (*P < 0.05).

The antibacterial effects of Si_3_N_4_ samples prepared by different sintering processes and control samples on E. coli are shown in Fig. 5a–g. The bacterial count of corresponding flat plates for Al_2_O_3_ (a), Ti-alloy (b), PEEK (c), MS Si_3_N_4_ (d), GPS Si_3_N_4_ (e), and HPS Si_3_N_4_ (f) were 68 ± 10, 35 ± 7, 79 ± 13, 14 ± 5, 21 ± 5, and 25 ± 4, respectively. The bacterial count of the GPS Si_3_N_4_, HPS Si_3_N_4_ and MS Si_3_N_4_ group is much smaller than that of PEEK and Al_2_O_3_, and slightly smaller than that of Ti-alloy. This indicated the antibacterial activity of Si_3_N_4_ prepared by three sintering processes against E. coli is stronger than the other three control samples. Among the three sintering processes, MS Si_3_N_4_ has the strongest antibacterial activity.

The antibacterial effect of Si_3_N_4_ samples prepared by different sintering processes and control samples on *S. aureus* is shown in Fig. 5A–G. It can be clearly seen that the bacterial count of the Si_3_N_4_ group sintered by GPS, HPS, and MS is much smaller than that of Al_2_O_3_, Ti-alloy, and PEEK, and PEEK has the highest residual bacterial count. The bacterial count of corresponding flat plates for Al_2_O_3_ (A), Ti-alloy (B), PEEK (C), MS Si_3_N_4_ (D), GPS Si_3_N_4_ (E), and HPS Si_3_N_4_ (F) were 49 ± 9, 55 ± 8, 67 ± 11, 7 ± 5, 10 ± 6, and 13 ± 8, respectively. The bacterial count of the GPS Si_3_N_4_, HPS Si_3_N_4_, and MS Si_3_N_4_ group is much smaller than that of Al_2_O_3_, Ti-alloy, and PEEK. The antibacterial activity of Si_3_N_4_ samples prepared by three sintering processes against *S. aureus* is stronger than the other three control samples. Among the three sintering processes, MS Si_3_N_4_ has the strongest antibacterial activity.

### Cell adhesion and morphology

**Figure 6:**
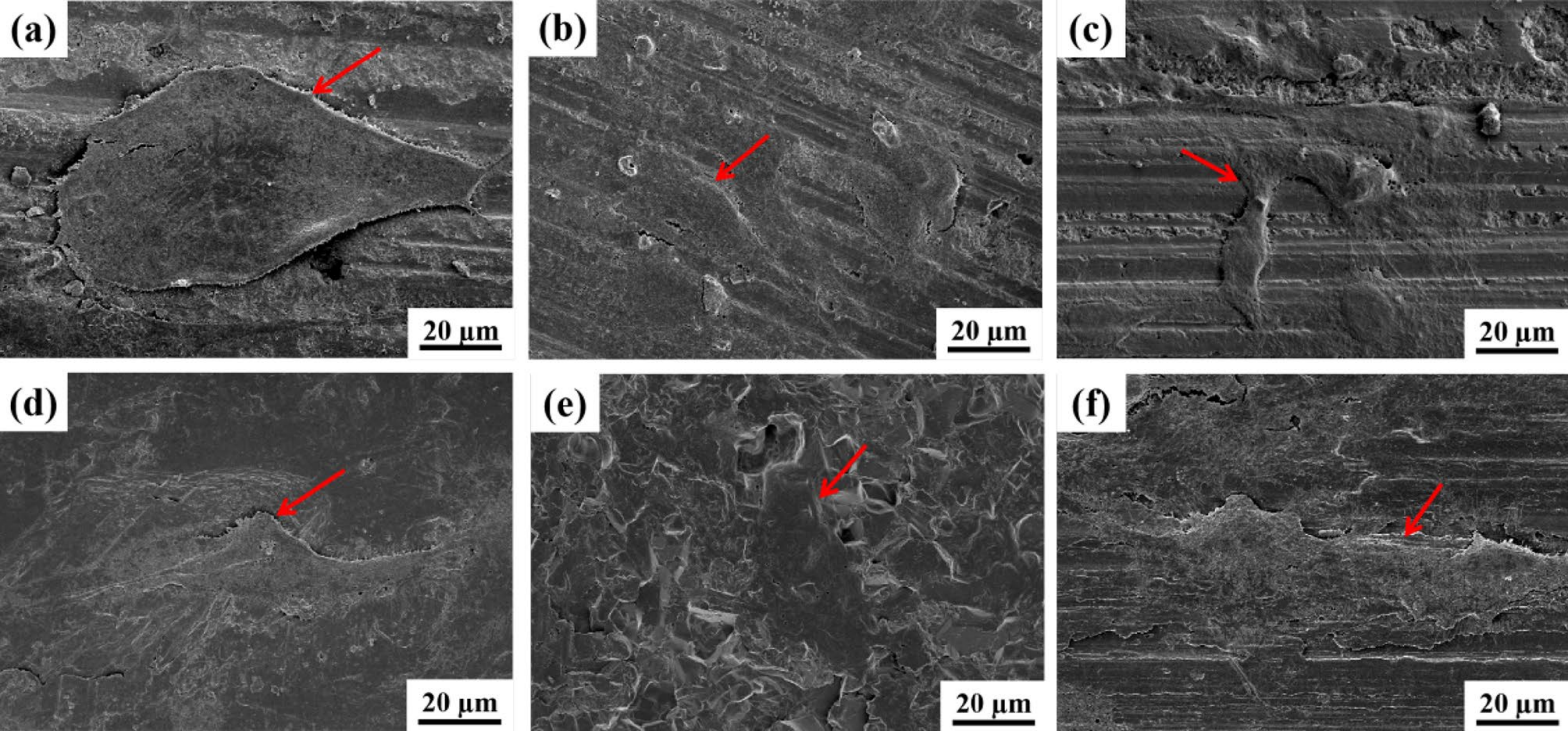
Cell adhesion of different materials: (**a**) MS Si_3_N_4_; (**b**) GPS Si_3_N_4_; (**c**) HPS Si_3_N_4_; (**d**) PEEK; (**e**) Al_2_O_3_; (**f**) Ti-alloy. (Red arrow: cell).

The cell adhesion on different materials is shown in Fig. 6. As shown in Fig. 6, cells can adhere to various materials and exhibit a patchy distribution on all materials. In the electron microscope, the adhesion morphology of cells changes due to the surface morphology of the material. From Fig. 6a–c, on the surfaces of the three types of Si_3_N_4_, cells mainly adhere to the materials in spindle or divergent shapes. At the same time, filamentous pseudopodia extending from the cell edge to the material surface can be observed. Among them, the cells on the MS sample adhered tightly and maintained good morphology, followed by the GPS sample, which adhered tightly but had weak morphology, and the HPS sample had poor adhesion and dispersed morphology. From Fig. 6d–f, the adhesion of cells on PEEK, Al_2_O_3_, and Ti-alloy is different from that on Si_3_N_4_. Due to the influence of surface morphology and material, the cells cannot maintain their original morphology, which has a certain impact on their adsorption, migration, and reproduction.

**Figure 7:**
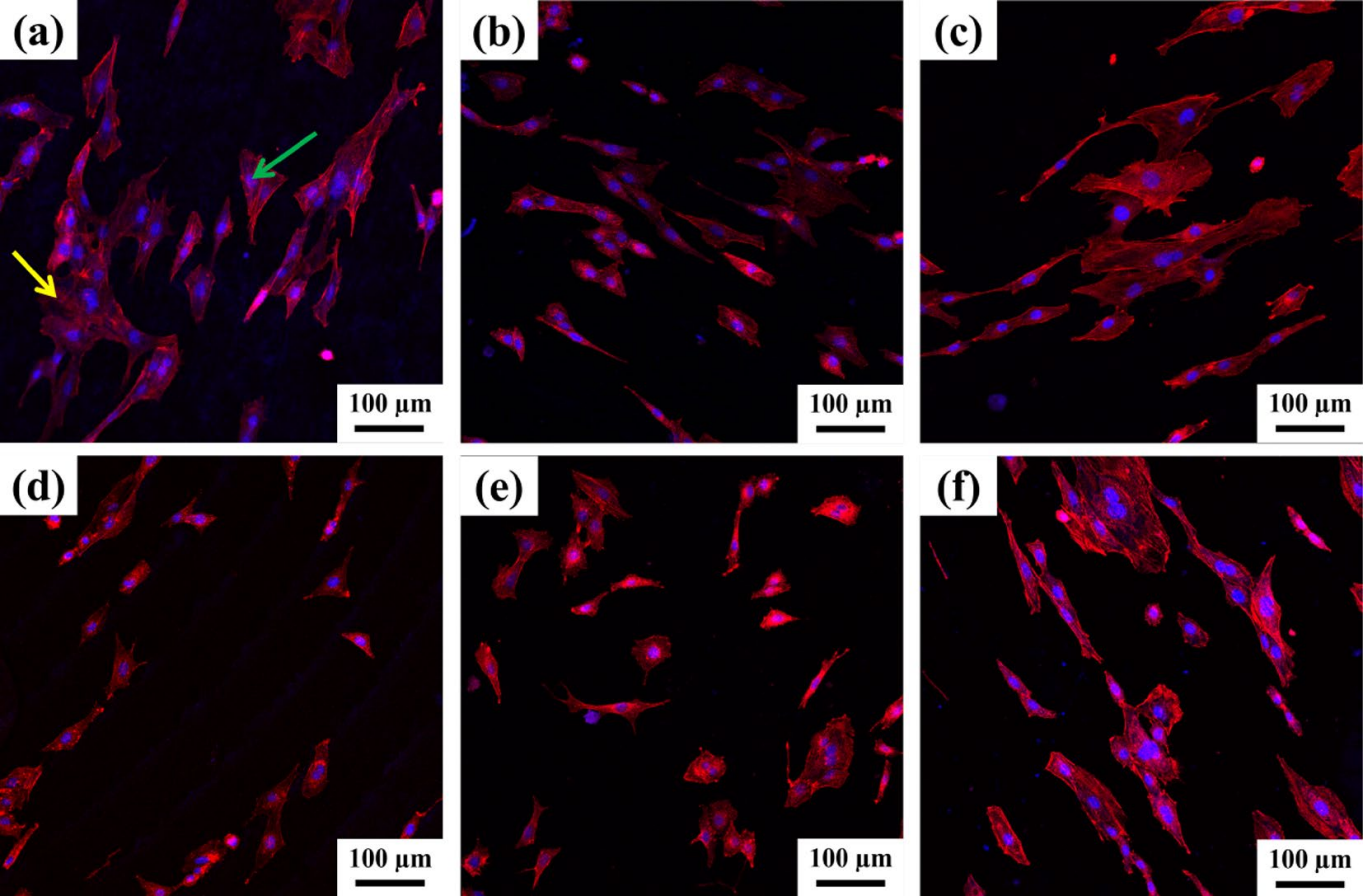
Morphology of Si_3_N_4_ cell cytoskeleton on different material surfaces: (**a**) MS Si_3_N_4_; (**b**) GPS Si_3_N_4_; (**c**) HPS Si_3_N_4_; (**d**) PEEK; (**e**) Al_2_O_3_; (**f**) Ti-alloy. (Green arrow: nucleus, yellow arrow: actin filament).

Stain the cells on the material using Actin-Tracker Red-Rhodamine and DAPI, and observe with CLSM, as shown in Fig. 7. It can be observed that the cell skeletons on the surfaces of the three Si_3_N_4_ materials all exhibit divergent or spindle like shapes, but their specific shapes, quantities, and sizes vary to some extent. The cell skeleton morphology on the MS samples is the best. In addition, the cells on the three types of Si_3_N_4_ are cross-linked with each other, and the red microfilament proteins of each cell bind to each other. The cell skeletons on PEEK, Al_2_O_3_, and Ti-alloys exhibit varying sizes, slightly fewer quantities, and irregular shapes. From the number of blue circular spots and red microfilament protein connections on each sample, it can be inferred that the number or degree of cell connections in the three types of control materials are weaker than those in the three types of Si_3_N_4_ materials.

## Discussion

Si_3_N_4_ material is the “king of comprehensive performance” in inorganic materials, with excellent comprehensive performance and great application prospects in many fields^36^. However, Si_3_N_4_ material has the characteristics of high sintering temperature and difficult sintering density^32,37^. When the sintering temperature exceeds 1600 °C, the higher the temperature, the more severe its volatilization and decomposition^32^. Even with high nitrogen pressure, the high-temperature decomposition of Si_3_N_4_ material cannot be avoided, Traditional sintered Si_3_N_4_ materials generally adopt the method of HPS to apply greater mechanical pressure to promote the dense sintering of Si_3_N_4_, or adopt the method of GPS to add more liquid phase sintering additives to promote the dense sintering of Si_3_N_4_^38^. In the HPS method, due to the special nature of mechanical pressure applied from top to bottom, only flat and simple products can be prepared, which has significant limitations on the shape of the products and is not suitable for sintering complex shaped products ^39^. In the GPS method, in order to achieve high density, more liquid phase sintering aids are added or the sintering temperature is increased to improve the sintering ability of the liquid phase, which promotes the ability of α-Si_3_N_4_ converted into β-Si_3_N_4_ phase. It leads to the presence of a more obvious glassy second phase substance at the grain boundaries of Si_3_N_4_ sintered body, which has a certain effect on the mechanical performance of the material^40^. Moreover, traditional sintering equipment such as GPS furnaces and HPS furnaces have heating elements, fixtures, carriers, insulation layers, etc. made of graphite or carbon materials. At high temperatures, there is a large amount of carbon thermal reduction atmosphere in the sintering chamber that is not controlled, resulting in chaotic and turbulent sintering atmosphere. Moreover, at high temperatures, the carbon thermal reduction atmosphere is prone to interface reaction with Si_3_N_4_ materials, generating silicon carbide (SiC) and nitrogen, causing corrosion and infiltration on the surface of Si_3_N_4_ material, resulting in the decrease in material properties^41^. Therefore, traditional sintering methods such as GPS and HPS have the significant effect on the sintering properties of Si_3_N_4_ materials due to the unstable sintering environment. In addition, due to the fact that both HPS and GPS belong to external heating, the heating element heats up the Si_3_N_4_ material sample through thermal conduction or radiation. Therefore, there exists a local process of heating first and then gradually from the outside to the inside, which not only makes the degreasing process long-time, has multiple procedures, and has a high probability of defects, but also leads to inconsistent and uneven time for the liquid phase generation of internal sintering aids, resulting in irregular crystal structure growth under the action of the liquid phase during the Si_3_N_4_ crystal phase transformation process, which has the significant effect on material performance^42,43^.

$$Si_{3} N_{4} + 3C \to 3SiC + 2N_{2}$$

In this research, compared with GPS Si_3_N_4_ and HPS Si_3_N_4_, MS Si_3_N_4_ have ideal microstructure and excellent mechanical properties, which are closely related to the characteristics of MS. MS is a green sintering technology that relies on the material itself to absorb microwave energy at high-frequency microwave frequencies to achieve self-heating and heating^25,28^. In this research, four 1.5 kW 2.45 GHz microwave sources were used in the microwave sintering equipment, which were uniformly arranged in a three-dimensional manner. At the same time, the insulation barrel inside the microwave cavity always maintained a clockwise rotation state of 60 revolutions per minute. Therefore, under the combined effect of microwave uniform radiation and effective diffuse reflection, uniform heating of the material was achieved, which is conducive to achieving the same frequency resonance of the material itself and completing high-quality degreasing and the sintering densification process quickly and effectively, beneficial for high-quality growth and complete growth of silicon nitride crystals, and beneficial for improving material properties^44^. In terms of microwave sintering structure, due to the selective heating characteristics of microwaves, the research used recrystallized SiC material as an auxiliary heating plate and constructed a microwave hybrid heating technology sintering structure. By utilizing the excellent microwave absorption performance of recrystallized SiC materials at low temperatures and the effective absorption of microwaves by organic binders (The thermal decomposition carbon products of PE and PW) in the 3D printed bodies, effective and uniform auxiliary heating was carried out when the absorption performance of Si_3_N_4_ materials was poor at lower temperatures^25^. After the sintering temperature reached 600 °C, the absorption performance of Si_3_N_4_ material was significantly improved. At this time, utilizing the characteristic of relatively small changes in the absorption performance of recrystallized SiC materials at high and low temperatures, the absorption performance of recrystallized SiC material is still at the original level at high temperatures. Therefore, the main body that absorbs microwave energy gradually realizes the transformation from recrystallized SiC materials to Si_3_N_4_ materials^45^. Finally, the densification sintering of Si_3_N_4_ materials is mainly achieved through the absorption of microwave energy by the Si_3_N_4_ materials themselves.

At the 2.45 GHz high-frequency microwave frequency, the sintering activity of Si_3_N_4_ material was significantly improved, the liquid phase generation temperature was significantly reduced, the dense sintering temperature of Si_3_N_4_ material was significantly reduced, and the high-temperature decomposition and volatilization of Si_3_N_4_ were effectively suppressed. Therefore, in this study, microwave sintering of dense silicon nitride materials was achieved at only 1620 °C and did not require gas pressure or mechanical pressure to promote densification sintering. Compared with the HPS temperature of 1650 °C and the GPS temperature of 1700 °C, the phenomenon of volatilization and decomposition of samples during microwave sintering is significantly reduced. The overall self-heating method of Si_3_N_4_ material effectively ensures the high uniformity of material heating, the uniform removal of organic binders, the uniform generation of liquid phase, and the uniform transformation of Si_3_N_4_ crystal phase^42^. During the crystal phase transformation, the improvement of the uniform liquid phase effect allows the Si_3_N_4_ crystal to grow fully, forming a more perfect and ideal Si_3_N_4_ crystal structure with longer diameter, and the comprehensive mechanical performance of Si_3_N_4_ material has been significantly improved^46^. In addition, due to the special structure of microwave equipment, high-temperature volatiles are cooled and adhered to the water-cooled furnace wall after coming out of the insulation barrel, completely isolated from the high-temperature sintering area. The high-temperature sintering area is surrounded by insulation barrels layer by layer, and the temperature outside the insulation barrel is about 60–80 °C. The distance between the insulation barrel and the metal water-cooled furnace wall is more than 50 cm, and the temperature of the metal water-cooled furnace wall is around 20 °C under normal pressure, The high-temperature volatile atmosphere will not affect the sintering atmosphere in the high-temperature sintering zone, and there is no graphite or carbon material in the MS structure, so there will be no carbon reduction atmosphere in the GPS and HPS, thereby ensuring a pure atmosphere in the high-temperature sintering zone. It has significant gain impact on the high-quality sintering of silicon nitride materials and the ideal growth of Si_3_N_4_ crystals, thereby achieving excellent mechanical properties. This is also the main reason why MS Si_3_N_4_ materials have significant advantages in biocompatibility, cytotoxicity testing, evaluation of antibacterial properties, and comparison of cell adhesion and morphology.

**Figure 8:**
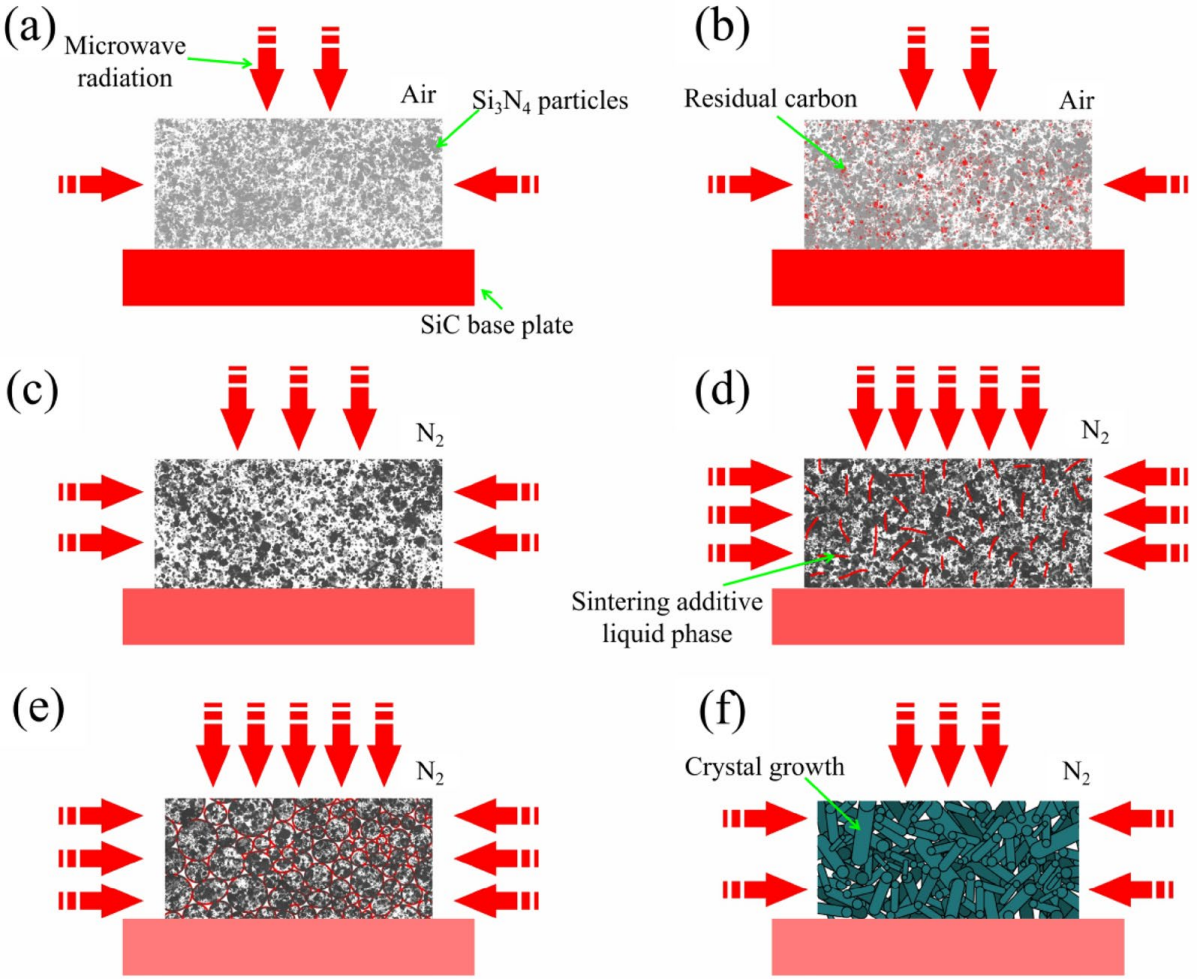
Schematic diagram of the principle of microwave sintering 3D printing of Si_3_N_4_ material: (**a**) Si_3_N_4_ particles and sintering additives are 3D printed into a green body, which is then placed in a microwave sintering furnace for degreasing in the air. The SiC substrate absorbs microwave energy to assist in heating the Si_3_N_4_ green body; (**b**) The microwave radiation heating of Si_3_N_4_ green body decomposes organic matter into carbon products to assist in heating Si_3_N_4_ green body, and gradually oxidizes and removes it in the air; (**c**) As the temperature continues to rise, the microwave absorption capacity of Si_3_N_4_ material significantly increases, while the microwave absorption capacity of SiC substrate relatively decreases; (**d**) When the temperature reaches a certain level, a sintering additive liquid phase is generated, which slowly wets the Si_3_N_4_ particles and further enhances the overall microwave absorption ability of the Si_3_N_4_ body; (**e**) The liquid phase effect of the additive is enhanced, gradually enveloping the Si_3_N_4_ particles. The Si_3_N_4_ crystal phase begins to transform, and the ability of the SiC substrate to absorb microwaves is further reduced; (**f**) Si_3_N_4_ gradually undergoes phase transition under the action of liquid phase, forming a columnar shape β-Si_3_N_4_ crystal. The distance between crystals decreases, and the liquid phase slowly evaporates, resulting in a significant reduction in the volume of the material. The material gradually becomes dense and its ability to absorb microwave energy decreases.

Due to the decrease in liquid phase generation temperature of Si_3_N_4_ materials in microwave environments, the densification and sintering temperature of Si_3_N_4_ materials can be significantly reduced^47^. Low temperature sintering of dense Si_3_N_4_ materials with small grains helps to increase the comprehensive mechanical performance of Si_3_N_4_ materials^48^. From the appearance after sintering, it can be seen that the sample sintered by microwave is not affected by the carbon atmosphere, and the color is significantly lighter. From the microscopic morphology, the micro grain boundaries of the sample sintered by microwave are clean. This is because during the sintering process, the Si_3_N_4_ body reaches the liquid phase temperature and forms a glassy phase on the Si_3_N_4_ grain boundaries. After effectively assisting in the sintering of Si_3_N_4_ materials, it still has the characteristic of microwave selective heating, The liquid sintering aid continues to absorb a large amount of microwave energy, gradually evaporating and purifying, and finally obtaining a dense material of Si_3_N_4_ with clean grain boundaries^49,50^. The schematic diagram of MS principle is shown in Fig. 8a–f. At different stages of MS, the degree to which samples absorb microwaves varies. At low temperatures, Si_3_N_4_ materials absorb less microwaves and rely on auxiliary heating materials to absorb microwaves to increase temperature. Auxiliary heating materials include recrystallized SiC materials and organic binders (The thermal decomposition carbon products of PE and PW)^25^. After reaching a certain temperature, especially after the liquid phase appears, the microwave absorption ability of Si_3_N_4_ materials is greatly improved, while the microwave absorption ability of auxiliary heating material SiC is relatively reduced, and the microwave energy is concentrated in the Si_3_N_4_ material itself. In the later stage of sintering, the liquid phase gradually decreases, and the absorption ability of silicon nitride materials decreases. At the same time, Si_3_N_4_ materials gradually complete dense sintering. The dense sintering mechanism of Si_3_N_4_ is a process of transformation from α phase to β phase^51^, which is achieved in the liquid phase. At a certain temperature, due to the action of additives, a liquid phase is generated that is close to the Si_3_N_4_ crystal^52^. The Si_3_N_4_ crystal phase dissolves and precipitates in the liquid phase, completing the transformation and densification process of the Si_3_N_4_ crystal phase^3^. At the same time, the liquid phase slowly evaporates, and the spacing between crystals decreases^53^. The ideal state is complete evaporation of the liquid phase, Si_3_N_4_ crystals are tightly bound in a directional manner, with pure and impurity free phases at grain boundaries, accompanied by significant macroscopic volume shrinkage^54^. Due to different sintering methods, harsh atmosphere and inconsistent temperature uniformity, the additive effect is poor, the densification process is not ideal, the crystal growth is incomplete, the crystal growth direction is disordered, and finally the Si_3_N_4_ crystals cannot be perfectly tightly combined, which has significant negative effect on the material properties^7,55^. The columnar crystals of β-Si_3_N_4_ are the main reason for the high mechanical properties of Si_3_N_4_ materials, so the diameter of crystals, aspect ratio, and the integrity of crystal growth, are important factors determining whether Si_3_N_4_ materials can have high mechanical properties^56^. Among them, the aspect ratio and directionality of the crystal are more critical^57^. From the experimental results of this study, it can be seen that compared to GPS Si_3_N_4_ and HPS Si_3_N_4_, MS Si_3_N_4_ yields a suitable diameter size of the columnar crystal achieves high mechanical properties due to its high aspect ratio, intact crystal morphology, and directionality.

## Conclusions

This study successfully prepared 3D printed dense Si_3_N_4_ material using a newly constructed microwave heating structure. At present, the mechanical performance and biological activity of Si_3_N_4_ materials aim to test whether the basic properties of microwave sintered 3D printed Si_3_N_4_ materials as bone replacement materials meet the basic requirements of applications. Compared with Alumina, Titanium alloy, PEEK, GPS Si_3_N_4_ and HPS Si_3_N_4_, the 3D printed Si_3_N_4_ bioceramics sintered by microwave have the best mechanical performance: bending strength is 928 MPa, fracture toughness is 9.61 MPa·m^1/2^, vickers hardness is 16.2 GPa, compressive strength is 3215 MPa. Meanwhile, it has excellent biocompatibility and antibacterial properties, and the activity of cells on its surface is excellent. Due to the special microwave sintering effect, a large amount of organic matter in 3D printed Si_3_N_4_ bodies can be efficiently and high-quality removed, the β-Si_3_N_4_crystal growth is more complete, the crystal diameter is smaller, and the crystal aspect ratio is larger, significantly improving the mechanical properties and vitro biological activity of Si_3_N_4_ materials. Microwave-sintered 3D-printed Si_3_N_4_ bioceramics have significant properties advantages and are an excellent and promising substitute material for implants. This research provides a feasible preparation program for the customization and high reliability performance of silicon nitride bioceramic. Such results should provide better clinical outcomes, improve the patient's medical experience and quality of life.

### Supplementary Information

Supplementary Video 1.

## Data Availability

All data generated or analysed during this study are included in this published article.
